# Comorbid depression and anxiety: Integration of insights from attachment theory and cognitive neuroscience, and their implications for research and treatment

**DOI:** 10.3389/fnbeh.2022.1104928

**Published:** 2022-12-21

**Authors:** Ravi Philip Rajkumar

**Affiliations:** Department of Psychiatry, Jawaharlal Institute of Postgraduate Medical Education and Research (JIPMER), Puducherry, India

**Keywords:** depression, anxiety disorders, comorbidity, attachment theory, theory of mind, neuropeptides, somatic markers, insecure attachment

Depressive and anxiety disorders are the commonest mental disorders worldwide. These disorders are highly comorbid with each other. A number of biological and psychological processes have been invoked to explain this degree of comorbidity, but these mechanisms require integration into an empirically verifiable explanatory model that would guide research and therapeutic strategies. Attachment theory provides a theoretical framework to account for the co-occurrence of anxiety and depression, as part of the triphasic response to separation. Early childhood attachment can be classified as secure or insecure. Insecure attachment to caregivers can predispose to the development of both anxiety and depression in adult life, particularly in response to interpersonal or social stressors. Cognitive neuroscience provides a complementary approach to understanding this comorbidity, based on altered information processing in specific brain circuits. Contemporary research has deepened our understanding of the neurobiological correlates of insecure attachment, and has permitted the identification of a substantial overlap between the constructs of attachment theory and the empirical findings of cognitive neuroscience. More specifically, it is now possible to outline the manner in which insecure attachment leads to alterations in higher-order cognitive, affective and social processes that predispose to both depression and anxiety. In this paper, I highlight findings linking attachment theory and cognitive neuroscience in patients with comorbid anxiety and depression, outline the causal pathways involved, and discuss the manner in which these insights can lead to improved strategies for prevention, early intervention, and more effective treatment of this pattern of comorbidity.

## Introduction

### A. Comorbid depression and anxiety: An overview

Depressive and anxiety disorders are the commonest mental disorders worldwide. Estimates from the Global Burden of Disease Study (2019) suggest that over 279 million people suffer from depressive disorders, and over 301 million suffer from anxiety disorders (GBD 2019 Mental Disorders Collaborators, 2022). In addition to their high prevalence, depression and anxiety disorders are highly comorbid with each other. A meta-analysis of over 170 published studies suggests that the median odds ratio for comorbidity between these disorders is over 6: in other words, individuals with one group of disorders are six times more likely to have the other disorder than those without either diagnosis (Saha et al., 2021). The presence of comorbid depression and anxiety has significant implications in terms of the course and outcome of both disorders: people with comorbid depression and anxiety tend to have an earlier age at onset of either illness, higher rates of childhood trauma, higher levels of neuroticism, more severe functional impairment, and a poorer treatment response (Hung et al., 2020; Breteler et al., 2021; ter Meulen et al., 2021).

A number of biological mechanisms have been proposed to account for this comorbidity, including shared genetic architecture (Cerda et al., 2010), alterations in immune-inflammatory pathways (Gaspersz et al., 2018), dysregulation of the hypothalamic-pituitary-adrenal (HPA) axis (Vreeburg et al., 2013; Steudte-Schmiedgen et al., 2017), abnormalities of the gut-brain axis (Simpson et al., 2021), and altered functioning of specific brain regions such as the frontal and temporal cortices, insula, amygdala and basal ganglia (Nawjin et al., 2022; Sindermann et al., 2022). From a psychosocial perspective, this comorbidity has been seen as reflecting a shared temperamental diathesis (Jeronimus et al., 2016), exposure to specific risk factors in childhood or later life (Teicher and Samson, 2013; van Tol et al., 2021), or a combination thereof. From a neuroscientific perspective, comorbid anxiety and depression can be understood in terms of dysfunction of specific brain circuits or modules involved in the resolution of short- and long-term goal conflicts. These modules are themselves influenced by both genetic and environmental factors (McNaughton and Corr, 2016).

From a nosological as well as a therapeutic perspective, an overarching theoretical model that could integrate these findings is required. Such a model would facilitate the development of novel approaches in the treatment of comorbid depression and anxiety, and overcome the limitations inherent in existing categorical classifications (Demyttenaere and Heirman, 2020; Tanaka et al., 2022a,b,c). This paper begins by examining the potential utility of attachment theory, as developed by John Bowlby and his collaborators and refined through subsequent decades of research, as one such integrative model. Next, the complementary perspective provided by cognitive neuroscience approaches to comorbid depression and anxiety is outlined, followed by the findings of clinical and neurobiological research that suggest an overlap between these two perspectives. Finally, a proposal for the integration of attachment and cognitive neuroscience findings in this patient population is outlined, and its implications for research, prevention and treatment are discussed.

### B. Attachment theory and cognitive neuroscience

Attachment theory was originally formulated by John Bowlby in response to the limitations of psychodynamic approaches to the management of anxiety and depression. Thus, from its conception, this model was intended to address the development and management of these conditions. According to attachment theory, behavior aimed at maintaining proximity to an attachment figure—usually the mother—from the earliest stage of life is a pre-programmed, evolutionarily conserved process whose primary function is protection from predators or other dangers (Bowlby, 1988). In addition to this primordial function, attachment in humans also serves the purpose of forming neural representations (“internal working models”) of the self, others, and the relationships between them, thereby influencing the development of higher-order processes such as emotion regulation, inhibitory control, communication skills, and social behavior (Bowlby, 1998; Laurita et al., 2019; Kamza and Putko, 2021). Separations from an attachment figure in early life, most typically between the ages of 6 months and 3 years, evoke a typical tripartite response: an initial phase of anxiety and anger, a subsequent phase of sadness or depression, and finally a period of “detachment” if separation is unduly prolonged or severe (Bowlby, 1988). The process of attachment does not depend exclusively on the caregiver's responses to the child, but is also influenced by the child's temperament (Raby et al., 2012). Childhood temperament is determined both by genetics and by pre- or perinatal factors influencing brain development (Takegata et al., 2021). Over time, individuals develop a typical “attachment style” which may be either “secure” or “insecure”, and this style forms a cognitive, affective and behavioral template for subsequent relationships in adolescence or adulthood (Shahab et al., 2021). In the initial work of Bowlby and Ainsworth, attachment was classified as “secure” or “insecure” based on the child's observed response to temporary separation from their caregiver under controlled conditions (Voges et al., 2019). Subsequent research led to identification of three subtypes of insecure attachment in childhood: anxious-avoidant, anxious-ambivalent, and disorganized (Ainsworth, 1985; Cameron, 2008). Research in adults has also identified three distinct “styles” of insecure attachment in adults: preoccupied, dismissing and disorganized (Ward et al., 2006). There is evidence of significant continuity between childhood and adult attachment (Ammaniti et al., 2000; Lewis et al., 2000; Platts et al., 2005). An overview of attachment patterns in childhood and adulthood, and their continuity, is provided in Table 1.

**Table 1:** Attachment styles in childhood and adulthood.

**Childhood attachment pattern**	**Description**	**Corresponding adult attachment style**	**Description**
Secure	Exploratory behavior using the caregiver as a “secure base”; self-limited and appropriate distress on separation; preference for caregiver over strangers.	Secure	Normal self-esteem; stable relationships; good ability to communicate needs and emotions and to respond to others' needs
Anxious-avoidant	Little emotion expressed on separation; diminished response to reunion; reduced caregiver preference.	Dismissive (avoidant)	Exaggerated sense of self-sufficiency; avoidance of close relationships; difficulty in communicating emotions or needs
Anxious–ambivalent	Anxiety even before caregiver leaves; prolonged distress on separation; expresses both anxiety and anger to caregiver on reunion.	Preoccupied (anxious)	Significant baseline anxiety; low self-esteem; fear of abandonment; high levels of anxiety on real or threatened separation; often seeks approval of significant others
Disorganized	No clear pattern of responses; behavior around caregivers odd or inconsistent (aggression, “reversal” of caregiver role, turning away from caregiver); may treat caregivers and strangers equally. Reflects significant degrees of abuse or neglect.	Disorganized (fearful-avoidant)	Unstable or chaotic relationships; low self-esteem; difficulties in emotion regulation

**Figure 1:**
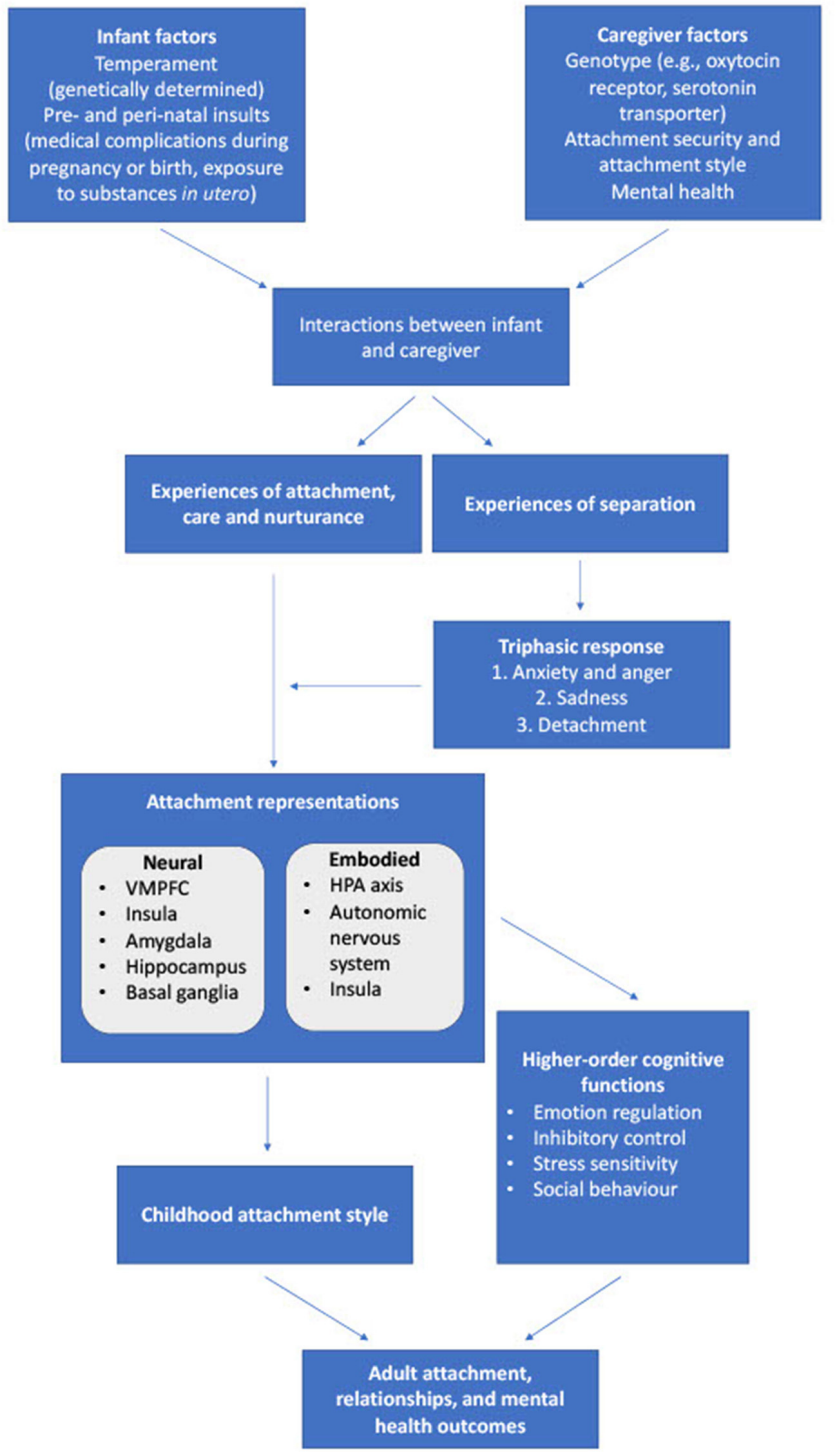
Processes involved in childhood attachment, their physiological correlates, and their effects on cognition and adult outcomes. VMPFC, ventromedial prefrontal cortex; HPA, hypothalamic-pituitary-adrenal.

Recent research has shed much light on the neural and molecular correlates of attachment and its relationship to higher-order cognitive processes. A particularly interesting development is the possibility that attachment representation and styles may be partially or completely embodied. This means that experiences of secure or insecure attachment are associated with alterations in sensory input (such as physical contact with a caregiver) which interact with perceptions of environmental threat *via* specific anatomical and chemical processes, leading to the activation of phylogenetically ancient behavioral systems including the stress response, attachment behavior, and exploratory behavior (Beckes et al., 2015). These lower-order somatic representations in turn influence both the formation of higher-order cognitive representations of the self and others, and the processing of information in social and interpersonal situations. If this process is optimal (secure attachment), then such “somatic markers” of attachment (Damasio, 1994) play an essential role in the development of subsequent social behavior, theory of mind, and stable interpersonal relationships (Long et al., 2020). On the other hand, if the process is suboptimal, higher-order information processing is inefficient or inaccurate, causing impairments in self-regulation and social behavior, and the maintenance of unstable or even abusive relationships (Miu et al., 2008; Kural and Kovacs, 2022), which predispose to the development of both depression and anxiety disorders. A graphical representation of the basic process of attachment and its neural correlates is provided in Figure 1.

It is notable that in his original work, Bowlby highlighted the congruence of attachment theory with cognitive theories of anxiety and depression, which were being outlined by the contemporary work of Aaron Beck (Bowlby, 1998; Beck, 2005). Thus, even in the 1960s and 1970s, when little evidence on the neurobiological correlates of attachment and its relationship to higher-order information processing was available, Bowlby envisaged a broader framework in which insecure attachment in childhood could explain the cognitive errors and biases seen in anxiety in depression (Bowlby, 1988, 1998).

Informed by the availability of neurobiological and translational evidence in subsequent decades, Beck and his colleagues discussed the manner in which cognitive models of depression and anxiety could be linked with this evidence, particularly as it pertains to emotional regulation and cognitive control in frontal and subcortical brain regions. This led to a cognitive neuroscience approach to comorbid depression and anxiety, in which cognitive errors were correlated with altered functioning of frontal-limbic circuits, which could potentially be reversed by cognitive therapy (Clark and Beck, 2010).

At this point in time, it is now possible to extend the cognitive neuroscience approach to anxiety and depression through its integration with attachment theory. Such a perspective, with is consistent with Bowlby's original vision, derives its support from research in translational models, as well as in healthy individuals and in those with anxiety and depression. For example, rodent models of early maternal separation and deprivation have been found to result in behaviors related to both anxiety and depression later in an animal's life (Rana et al., 2015; Frank et al., 2019). Likewise, it is known that representations of key figures from early childhood are neurally represented in an “attachment network” that includes the anterior cingulate and temporal cortices, insula, amygdala and thalamus (Ulmer-Yaniv et al., 2022) and that the level of anxiety associated with attachment is linked to altered functional connectivity between specific cortical areas (Deng et al., 2021). Moreover, it is increasingly clear that these representations of attachment shape processes such as social communication, social cognition and self-regulation (Jethava et al., 2022; Labek et al., 2022; Rogers et al., 2022). Impairments in these processes could increase an individual's vulnerability to both anxiety and depression, particularly when exposed to subsequent stress or adversity (Tanaka et al., 2022a). Finally, insecure attachment has been associated with increased systemic inflammation both in animal models and in humans (Ehrlich et al., 2019; Lumertz et al., 2022). Such a pro-inflammatory state can lead to symptoms of anxiety and depression both directly and through its effects on cognition (Carvalho et al., 2014; Tanaka et al., 2022b,c).

Before examining these mechanisms in more depth, it is first necessary to review the evidence for a link between disturbed attachment and the comorbidity between these disorders in clinical samples.

## Attachment and comorbid anxiety and depression: 1. Clinical evidence

According to Bowlby's original proposal, significant disruptions in attachment bonds occurring in early childhood would be associated with an increased risk of depression, while repeated experiences of temporary separation, or threats of separation, could predispose to anxiety (Bowlby, 1970, 1998; Holmes, 2013). Subsequent research suggests has allowed a better delineation of the effects of disrupted attachment on the clinical profile of these disorders. In depression, insecure attachment has been associated with greater symptom severity, a higher relapse rate, suicidal behavior, reluctance to seek help and poor adherence to medication (Ozer et al., 2015; Hooper et al., 2016; Conradi et al., 2018; Huang Y. C. et al., 2019; Cacciola and Psouni, 2020). In anxiety disorders, insecure attachment has also been associated with a poorer response to both pharmacological and psychological therapies (Milrod et al., 2016; Nielsen et al., 2019; Petrowski et al., 2019). Lower levels of secure attachment also appear to predict comorbid anxiety disorder in depressed patients (Marganska et al., 2013; Huang Y. L. et al., 2019). This association appears to be most specific for social anxiety disorder, in which insecure attachment is associated with both the presence and the severity of comorbid depression (Adams et al., 2018, 2019; Elling et al., 2022). These findings are consistent with a role for disturbances in childhood attachment in the onset, persistence and co-occurrence of depressive and anxiety disorders. The above studies, and other relevant observational research on insecure attachment, are summarized in Table 2. From these findings, it is clear that both preoccupied and avoidant patterns of adult attachment are associated with comorbid anxiety and depression, though there may also be an association with disorganized attachment when this pattern is associated with significant levels of fear. Having established this result with some consistency, it is now possible to examine the underlying cellular and neurobiological processes at work in patients with these disorders.

**Table 2:** Studies examining attachment patterns or styles in patients with comorbid depression and anxiety.

**Study**	**Sample size and characteristics**	**Results**
Eng et al. (2001)	Patients with social anxiety disorder (*n =* 118)	Anxious (preoccupied) attachment associated with more severe comorbid depressive symptoms.
Torpey et al. (2007)	Patients with depression with comorbid social anxiety disorder (*n* = 32), panic disorder (*n* = 41) or no comorbid anxiety disorder (*n* = 117)	Self-reports of lower perceived protection by a caregiver, suggestive of anxious attachment, were associated with comorbid panic disorder, particularly in men.
Marques et al. (2018)	Postpartum women (*n* = 450)	33.3% of women had significant symptoms of comorbid anxiety and depression; both preoccupied and avoidant attachment representations were more common in this sub-group.
Adams et al. (2018)	Patients with major depression (*n* = 44), major depression with comorbid social anxiety (*n* = 56), and healthy controls (*n* = 60)	Attachment anxiety and avoidance were both significantly elevated in patients with comorbid major depression and social anxiety compared to the other two groups.
Huang Y. C. et al. (2019)	Patients with major depression (*n* = 66); 74% of patients had a comorbid anxiety disorder, and 24% had multiple anxiety disorders	Secure attachment less common, preoccupied and avoidant attachment style more common in patients with comorbid anxiety; preoccupied style associated with multiple comorbid anxiety disorders.
Adams et al. (2019)	Patients with social anxiety disorder (*n =* 162)	Attachment avoidance and attachment anxiety were both positively associated with the severity of comorbid depressive symptoms.
Van Assche et al. (2020)	Elderly adults living in the community (*n* = 81) with no known diagnosis of anxiety or depression	Avoidant and preoccupied attachment styles were associated with higher levels of depressive and anxiety symptoms.
Radetzki et al. (2021)	Patients with major depression (*n =* 43), major depression with comorbid social anxiety (*n* = 56) and healthy controls (*n* = 60)	Self-reported attachment anxiety and avoidance were both significantly elevated in patients with comorbid depression and social anxiety.
Elling et al. (2022)	Patients with comorbid social anxiety and depression (*n* = 472) and social anxiety alone (*n* = 140)	Fearful (disorganized) attachment more common in patients with comorbid social anxiety and depression.

All findings refer to adult attachment pattern/style unless otherwise specified.

## Attachment and comorbid anxiety and depression: 2. Mechanisms

Insecure attachment also influences fundamental cognitive and affective processes implicated in the pathogenesis of anxiety and depression. These include impairments in emotion recognition (Monti and Rudolph, 2014), emotional regulation (Malik et al., 2015; Conrad et al., 2021), and theory of mind skills (Ozer et al., 2015; Koelkebeck et al., 2017). These deficits have been documented in patients with either disorder, as well as in patients with comorbid anxiety and depression (Marques et al., 2018; Radetzki et al., 2021). In addition, experiences of disrupted attachment appear to mediate the relationship between other environmental risk factors, such as childhood poverty or sexual abuse, and the subsequent risk of depression or anxiety (Briere and Jordan, 2009; Fearon et al., 2017).

The neural mechanisms underlying these higher-order processes are better understood now than they were in Bowlby's time. Attachment security appears to be encoded in both amygdalae (Lemche et al., 2006) and severe degrees of insecure attachment are associated with increased amygdalar volume (Moutsiana et al., 2015; Lyons-Ruth et al., 2016) and hippocampal volume (Hidalgo et al., 2019). Insecure attachment is also associated with altered functioning in the prefrontal, anterior cingulate, insular and parietal cortices, basal ganglia and amygdala during the performance of tasks related to social cognition or reward. The changes observed in these regions are suggestive of inefficient information processing and heightened reactivity to external situations (Warren et al., 2010; Moutsiana et al., 2014; Schneider-Hassloff et al., 2015; Quevedo et al., 2017; Miller et al., 2020). Avoidant and anxious attachment have both been associated with specific alterations in the functioning of a “social aversion circuit” involving the dorsal anterior cingulate cortex and middle temporal gyrus; such changes may be relevant to the fear of criticism or rejection, negative cognitions and avoidance behavior that are seen in patients with comorbid anxiety and depression (Krause et al., 2016). Insecure attachment representations have also been associated with increased asymmetrical activation of the parietal cortices when processing emotional memories, potentially leading to increased arousal, impaired emotion regulation, and an increased vulnerability to both anxiety and depression (Kungl et al., 2016). Unresolved attachment has also been associated with reduced white matter integrity in the corpus callosum, leading to cognitive impairments that could serve as a “general” vulnerability factor for both anxiety disorders and depression (Riem et al., 2019). The results of these imaging studies support the hypothesis of a close link between insecure attachment, altered or impaired cognition, and an increased vulnerability to comorbid anxiety and depression.

At a neurochemical level, these changes appear to correlate with alterations in dopaminergic circuitry (Strathearn, 2011) and peptidergic transmission, particularly involving endogenous opioid peptides and oxytocin (Muller et al., 2019). In particular, mu-opioid (μ) receptor availability has been found to correlate with adult attachment (Turtonen et al., 2020); lowered availability of these receptors has been associated with symptoms of both depression and anxiety (Nummenmaa et al., 2020). Likewise, oxytocin has been found to modulate neural circuits involved in fear as well as those involved in the processing of social information. As oxytocin is a key mediator of attachment behavior, insecure attachment could alter the influence of this transmitter on the activity and connectivity of brain regions such as the amygdala. This could be particularly relevant to the comorbidity between specific anxiety disorders, such as social anxiety disorder, and depression (Kirsch et al., 2005).

Insecure attachment has also been associated with alterations in autonomic nervous functioning, increased activity of the hypothalamic-pituitary-adrenal axis, and immune dysregulation (Jaremka et al., 2013; Abtahi and Kerns, 2017), all of which have been linked to the pathogenesis of comorbid depression and anxiety (McQuaid, 2021). Among these processes, alterations in cardiac autonomic functioning may be particularly important, as they represent a potential mechanism of “embodiment” through which experiences of attachment and separation can influence fear conditioning and sensitivity to both anxiety and depression (Battaglia and Thayer, 2022; Battaglia et al., 2022a,b; Gander et al., 2022). Cross-talk between central and peripheral structures involved in fear responses, such as the ventromedial prefrontal cortex, are also crucially influenced by attachment security (Eisenberger et al., 2011; Battaglia et al., 2022a). Likewise, the effects of attachment on the functioning of the HPA axis may explain the relationship between chronic stress and the onset of depression and anxiety in insecurely attached individuals (Smyth et al., 2015). The effect of attachment security on adult personality and mental health may itself be modified by genetic factors, such as functional polymorphisms of the oxytocin receptor (Schneider-Hassloff et al., 2016). There is also preliminary evidence that attachment security can influence the balance between pro- and anti-inflammatory cytokines; secure attachment stimulates the expression of anti-inflammatory genes (Stanton et al., 2017), while anxious attachment is associated with increased levels of inflammatory markers (Ehrlich et al., 2019). These changes could result in central nervous system inflammation and increased oxidative stress, providing another potential mechanism linking comorbid anxiety and depression with attachment (Tanaka and Vecsei, 2021; Tanaka et al., 2022b,c).

## Integrating attachment and cognitive neuroscience findings in comorbid depression and anxiety

**Figure 2:**
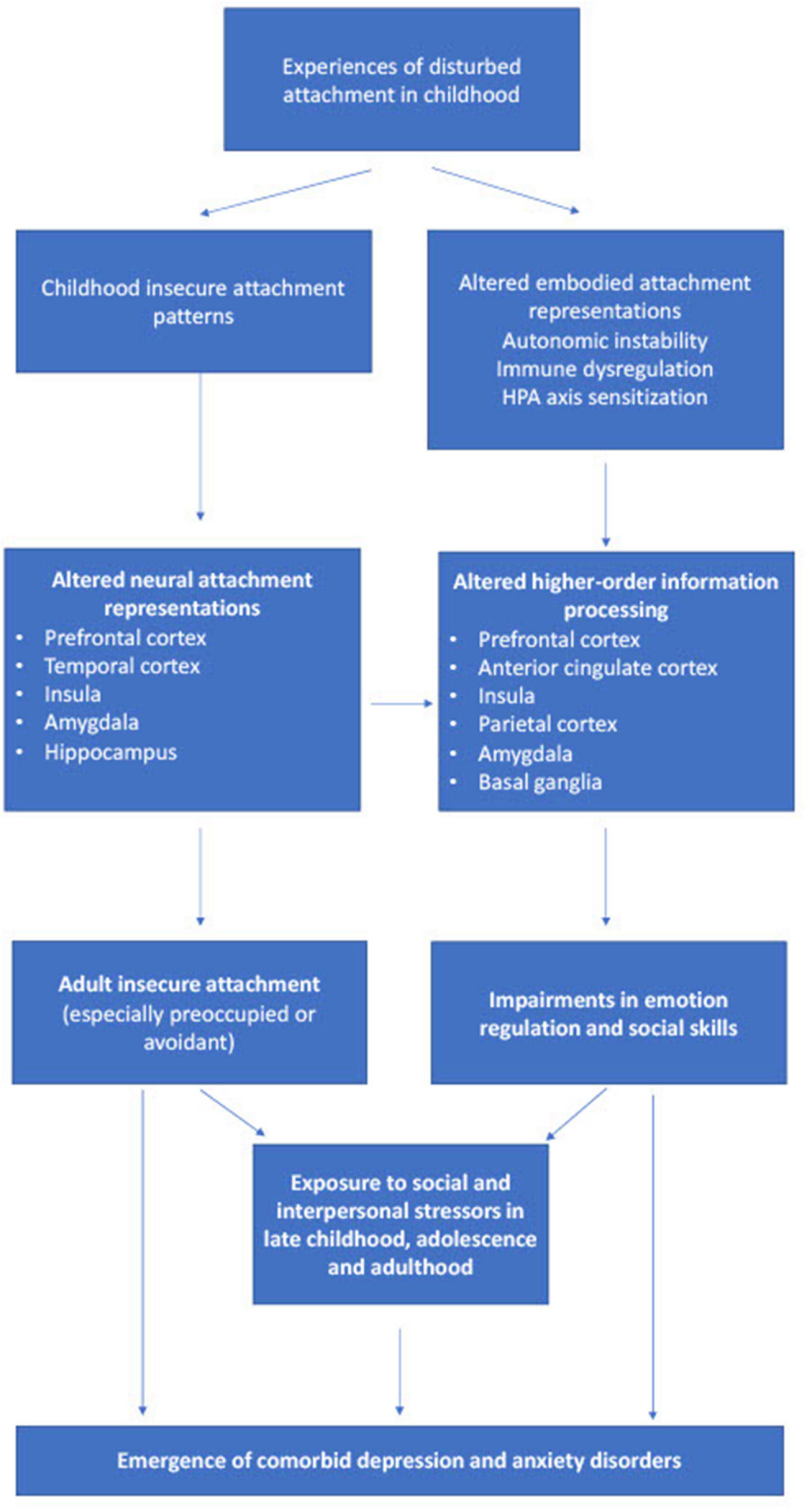
Processes by which insecure attachment to a caregiver in early childhood can contribute to the development of comorbid depression and anxiety, and their neural correlates. HPA, hypothalamic-pituitary-adrenal.

An integration of the above findings is presented in Figure 2. Early childhood attachment is determined by non-linear interactions involving several factors, including childhood temperament (itself the result of gene x environment interactions), parental attachment style and mental health, and broader socio-cultural factors (Barry et al., 2008; Toepfer et al., 2019; Takegata et al., 2021). Through processes that are likely to involve a combination of embodiment and higher-order representation, attachment influences the development of higher-order cognitive and affective processes related to the pathogenesis of anxiety and depression. If attachment is secure, the outcome of these processes is a secure adult attachment style and psychological resilience (Long et al., 2020). However, various patterns of insecure attachment can lead to inefficiency or biases in these higher-order processes, leading to insecure adult attachment, reduced relationship stability and satisfaction, and a sensitivity to both anxiety and depression, which are themselves components of the triphasic “separation response” (Bowlby, 1988, 1998). Also important in this model are the interactions between insecure attachment and other forms of adversity, which include social disadvantage, childhood abuse, discord or violence within adult intimate relationships, and other forms of chronic stress (Briere and Jordan, 2009; Fearon et al., 2017).

Though tentative, this proposal is consistent with our current understanding of the pathophysiology of anxiety (Nolte et al., 2011) and depression (Holmes, 2013). With the accumulation of further clinical and translational evidence, it should be possible to refine the above model in several ways. For example, the specific sub-type(s) of insecure attachment that predispose to comorbid anxiety and depression could be identified more consistently. Likewise, it is likely that distinct attachment-related neural and cognitive processes could be involved in the comorbidity between depression and specific anxiety disorders, such as social anxiety or panic disorder (Torpey et al., 2007; Elling et al., 2022). Finally, biomarkers related to insecure attachment, reflecting altered information processing in the brain circuitry involved in affect regulation, problem-solving and social cognition, could be identified in patients and assessed as predictors of outcome (Gander and Buchheim, 2015; van Hoof et al., 2019).

## Implications for research and treatment

The integration of perspectives and findings from attachment theory and cognitive neuroscience is of more than theoretical interest in patients with comorbid anxiety and depressive disorders. Such an approach could optimize treatment outcomes through the selection of attachment-based therapies that address either maladaptive attachment styles or the cognitive errors associated with them (Newman et al., 2015; Gunlicks-Stoessel et al., 2019; Cortes-Garcia et al., 2020). Such therapies could integrate existing psychodynamic or cognitive behavioral approaches under an attachment framework (Fonagy and Target, 2007; Holmes, 2013). For example, it has been shown that cognitive training focused on the modification of biases in information processing can modulate attachment anxiety (Doolan and Bryant, 2021). Couples or family therapies based on attachment theory may also be effective in treating adults with comorbid depression and anxiety, while minimizing the burden experienced by a spouse or partner (Johnson and Greenman, 2006). A better understanding of the neural and molecular mechanisms linking insecure attachment and comorbid anxiety and depression could also inform innovative approaches to biological treatment, such as pharmacological modulation of attachment-related peptidergic mechanisms (Kormos and Gaszner, 2013) or attachment-related alterations in immune or endocrine function (Hennessy et al., 2019), and even stimulation therapies that target attachment-related autonomic dysfunction (Fanselow, 2013). It should also not be assumed that the mechanisms discussed in this paper are the only ones linking attachment with anxiety and depression. For example, recent research suggests that experiences of attachment security may be “biologically embedded” in infants through alterations in DNA methylation (Merrill et al., 2021); such alterations involve changes in the expression of pro-inflammatory genes, which may be related to subsequent anxiety and depression.

Finally, pursuing this line of research could lead to a deeper understanding of the developmental roots of this comorbidity. It is well known that parental depression can affect infant attachment, and parental depression may itself reflect attachment patterns and experiences in the mother or father's own childhood (Sliwerski et al., 2020). More generally, patterns of attachment are known to be stable across generations, and this may reflect a bidirectional link with anxiety and depression: the presence of these disorders in a parent may both reflect their own childhood experiences and insecure attachment, and predict insecure attachment and subsequent anxiety or depression in their children (Galbally et al., 2022). Such an “intergenerational” transmission of attachment security has also been demonstrated in animal models, and been linked to altered expression of specific genes in the brain (Alyamani et al., 2021). A circular process of this sort offers opportunities for early intervention (e.g., identification and management of internalizing symptoms in children of depressed mothers) or even prevention (e.g., attachment-based therapies for parents whose depression and anxiety is related to attachment insecurity, thereby preventing the “transmission” of an insecure attachment style). Such an approach may be especially fruitful in “high-risk” families with an increased genetic risk for depression, or in families exposed to social disadvantage or chronic stress (Lecompte et al., 2018).

## Conclusion

Attachment theory offers a promising explanatory framework for our understanding of comorbid anxiety and depression. The coherence between the constructs of attachment theory and the findings of cognitive neuroscience, which was envisioned by early workers in this field, can now be placed on a more secure footing. The available evidence suggests that insecure attachment in childhood can crucially alter the subsequent functioning of key neurocognitive, neuroendocrine, and neuroimmune processes, leading to changes at both the physical level (such as increased inflammation and an altered stress response) and in higher-order cognitive functioning (cognitive errors and biases). These changes can predispose to the development and persistence of subsequent anxiety and depression. This framework may be heuristically useful both in terms of deepening our understanding of specific genetic, epigenetic, neural, immune and endocrine mechanisms, and in guiding the development of more effective treatment approaches, as well as opening up avenues for prevention and early intervention in childhood.

## Author contributions

The author confirms being the sole contributor of this work and has approved it for publication.
